# Association of metabolic syndrome with mobility in the older adults: a Korean nationwide representative cross-sectional study

**DOI:** 10.1038/s41598-021-86186-2

**Published:** 2021-03-23

**Authors:** Ki Young Son, Dong Wook Shin, Ji Eun Lee, Sang Hyuck Kim, Jae Moon Yun, Belong Cho

**Affiliations:** 1 Department of Family Medicine, Asan Medical Center, 88 Olympic-ro 43 gil, Songpa-gu, Seoul, 05505 Korea; 2 Department of Family Medicine/Supportive Care Center, Samsung Medical Center, Sungkyunkwan University School of Medicine, Seoul, Korea; 3 Center for Clinical Epidemiology, SAIHST, Sungkyunkwan University, Seoul, Korea; 4 Health Promotion Center, Seoul National University Hospital, Seoul, Korea; 5 Department of Family Medicine, Seoul National University Hospital, Seoul, Korea; 6 Department of Family Medicine, Bumin Hospital, Seoul, Korea; 7 Institute On Aging, Seoul National University College of Medicine, Seoul, Korea

**Keywords:** Metabolic syndrome, Geriatrics

## Abstract

We aimed to examine whether metabolic syndrome (MetS) is associated with mobility in the older adults, using the timed up and go (TUG) test which is one of the most widely used tests for evaluating mobility. This is population-based study with the National Health Insurance Service–National Health Screening Cohort database of National Health Information Database. Participants included were those who completed the TUG as part of the National Screening Program for Transitional Ages. An abnormal TUG result was defined as a time ≥ 10 s. Multiple logistic regression models were used to assess the associations between MetS and TUG results. We constructed three models with different levels of adjustment. Furthermore, we conducted a stratified analysis according to the risk. Among the 40,767 participants included, 19,831 (48.6%) were women. Mean TUG value was 8.34 ± 3.07 s, and abnormal TUG test results were observed in 4,391 (10.8%) participants; 6,888 (16.9%) participants were categorised to have MetS. The worst TUG test results were obtained in participants with three or four MetS features, and a J-shaped relationship of each MetS feature, except triglyceride (TG) and high-density lipoprotein-cholesterol (HDL-C), with TUG test was found. Participants with MetS had 18% higher likelihood of showing abnormal TUG test results in a fully adjusted model (adjusted odds ratio 1.183, 95% confidence interval 1.115–1.254). The stratified analysis revealed that participants with central obesity, high blood pressure, and normal HDL-C and TG were more likely to have abnormal TUG times. Participants with MetS had a higher risk of exhibiting abnormal TUG results, and except for HDL-C and TG, all other MetS features had a J-shaped relationship with TUG. Preventive lifestyle such as lower carbohydrate and higher protein intake, and endurance exercise is needed.

## Introduction

According to a report published by The World Health Organization, in 2012, cardiovascular (CV) disease was the leading cause of death by non-communicable diseases worldwide, and the number of deaths continues to increase^1^. Metabolic syndrome (MetS) is a cluster of health conditions, which are strongly associated with the incidence of CV diseases and mortality^2–4^. Its features include hyperglycaemia, raised blood pressure, elevated triglyceride (TG) levels, low high-density lipoprotein-cholesterol (HDL-C) levels, and central obesity. In Metabolic syndrome and Arteries REsearch consortium in Europe and the US, prevalence of MetS was 23.9% in men and 24.6% in women^5^. In Korea, the age-adjusted prevalence of MetS was 31.3% in 1998–2007^6^, and it increased to 36.1–38.6% in 2016^7^. And 12%–17% of the population develop CV disease associated with MetS^8^. Thus, currently, MetS is one of the most important public health problems in Korea.

The timed up and go (TUG) test is one of the most widely used tests among the tests for measuring mobility (e.g., Long distance corridor walk test, 6-min walk test, gait speed test, chair stand-up test). This test is easy to perform in clinical settings and can assess mobility, including static balance, dynamic balance, strength in the lower extremities, and gait speed. The test showed good sensitivity and specificity to identify frailty compared with other frailty measures^9^. Furthermore, TUG test can predict falls, fractures, and hospital admission^10,11^; disability and dependency^12,13^; dementia^14^; low quality of life and low social participation^15^; risk of CV diseases and mortality^16,17^; complications after elective surgery in cancer patients^18^; and onset of difficulty in performing activities of daily living (ADL) ^19^.

Previous studies have shown that a decline in mobility of older adults was associated with future CV disease and mortality^16,17,20–24^. Among them, two studies demonstrated the association of TUG test results with CV disease^16,17^. These findings implies that there is shared pathophysiology in mobility decline and CV disease in older adults. Thus, we hypothesised that TUG test results are associated with CV-related risk factors including MetS before CV events occur. Several studies examined the association of physical function with CV-related risk factors. These studies reported that MetS or some of its features or insulin resistance was associated with a decline in physical function^25–29^. Three such studies used TUG test as a measure of physical function^27–29^.

Although the previous studies revealed that abnormal TUG test results were associated with CV-related risk factors or MetS, the participants included in those studies were limited to the small population of patients with Parkinson’s disease or elderly women. To our knowledge, to date, no study has assessed these relationships in the large general populations comprising a sufficient number of MetS patients. Consequently, the generalizability of the relationship between MetS and TUG test becomes limited.

This study evaluated the association of MetS with TUG test results of 66-year-old participants selected from a large general population enrolled in the National Screening Program for Transitional Age (NSPTA) in Korea, which is a nationwide representative sample of Korean individuals.

## Results

### Baseline characteristics of participants

Of the 40,767 participants included, 19,831 (48.6%) were women. The mean height was 166.38 ± 5.55 cm for men, and 153.58 ± 5.29 cm for women, and the mean weight was 65.01 ± 8.46 kg for men, and 55.98 ± 7.54 kg for women. The mean body mass index was 23.64 ± 2.84 kg/m^2^. The mean values for TUG, waist circumference, HDL-C level, TG level, BP, and fasting glucose level were 8.34 ± 3.07 s, 81.6 ± 7.9 cm, 54.8 ± 21.2 mg/dL, 128.0 ± 77.8 mg/dL, 125.3 ± 14.8/76.7 ± 9.6 mmHg, and 97.7 ± 17.8 mg/dL, respectively; 4,391 (10.8%) participants showed abnormal TUG results (≥ 10 s), 9,531 (23.4%) participants had central obesity, 4,698 (11.5%) participants had a low HDL-C level, 10,910 (26.8%) participants had a high TG level, 18,218 (44.7%) participants had a high BP, and 13,887 (34.1%) participants had an impaired fasting glucose level.

Among the participants, 6,888 (16.9%) individuals were categorised to have MetS. Most participants with MetS (5,067, 73.4%) showed three MetS features, whereas only 212 (3.0%) of those participants showed all the five MetS features. Approximately, 20% of the participants reported about their depressive mood, and 15% were cognitively impaired (Table 1).

**Table 1:** Basic characteristics of participants.

	Total	Timed up and go	
		Normal	Abnormal
	N (%)	N (%)	N (%)
Sex (women) (%)	19,831 (48.6)	17,378 (47.8)	2,453 (55.9)
**Height (cm)**			
Men	166.38 ± 5.55	166.39 ± 5.54	166.23 ± 5.58
Women	153.58 ± 5.29	153.61 ± 5.27	153.28 ± 5.50
**Weight (kg)**			
Men	65.01 ± 8.46	65.03 ± 8.45	64.80 ± 8.61
Women	55.98 ± 7.54	55.91 ± 7.50	56.70 ± 7.82
Body Mass Index (kg/m^2^)	23.64 ± 2.84	23.61 ± 2.82	23.85 ± 3.01
Timed up and go test (s)	8.34 ± 3.07	7.65 ± 1.74	14.13 ± 5.03
Waist circumference (cm)	81.6 ± 7.9	81.6 ± 7.9	82.3 ± 8.1
HDL cholesterol (md/dL)	54.8 ± 21.2	54.8 ± 21.2	54.9 ± 21.0
Triglyceride (mg/dL)	128.0 ± 77.8	127.3 ± 77.3	134.0 ± 81.8
Systolic blood pressure (mmHg)	125.3 ± 14.8	125.2 ± 14.7	126.7 ± 15.3
Diastolic blood pressure (mmHg)	76.7 ± 9.6	76.6 ± 9.5	77.4 ± 10.0
Fasting glucose (mg/dL)	97.7 ± 17.8	97.6 ± 17.5	97.8 ± 20.0
**No. of metabolic syndrome feature**	1.4 ± 1.1	1.4 ± 1.1	1.5 ± 1.2
0	9,717 (23.8)	7,362 (24.5)	2,355 (21.9)
1	13,777 (33.8)	10,272 (34.2)	3,505 (32.6)
2	10.385 (25.5)	7,512 (25.0)	2,873 (26.8)
3	5,067 (12.4)	3,608 (12.0)	1,459 (13.6)
4	1,609 (4.0)	1,124 (3.7)	485 (4.5)
5	212 (0.5)	150 (0.5)	62 (0.6)
Metabolic syndrome	6,888 (16.9)	5,996 (16.5)	892 (20.3)
Depressive mood	7,480 (18.8)	6,400 (18.0)	1,080 (25.2)
Cognitive impairment	5,860 (14.5)	5,064 (14.2)	796 (18.6)

*HDL-C* high-density lipoprotein-cholesterol, *TG* triglyceride.

### Association of metabolic syndrome with timed up and go test

**Figure 1:**
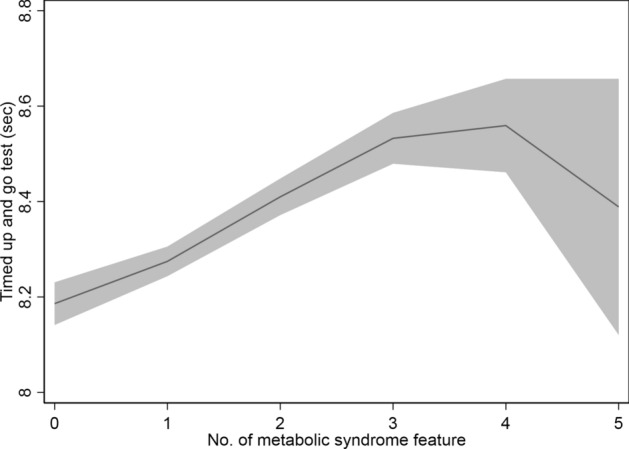
Association of Timed up and go test with the number of metabolic syndrome features. Gray area depicts 95% confidence interval.

An inverted U-shaped association was observed between the number of MetS features and TUG results. The worst TUG results were obtained in participants who had three or four MetS features rather than those who had five features (Fig. 1).

Among 33,879 participants without MetS, 3,499 (10.8%) participants had abnormal TUG results, whereas 892 (13.0%) participants with MetS showed abnormal TUG results.

In the crude model, participants with MetS were 18% more likely to have abnormal TUG results than participants without MetS (OR 1.183, 95% CI 1.117–1.253). Similarly, the participants had a 18% higher risk of showing abnormal TUG results in the fully adjusted model (adjusted OR (aOR) 1.183, 95% CI 1.115–1.254).

Similar results were found for both sexes. Men with MetS had a 15% higher risk of showing abnormal TUG result (aOR 1.146, 95% CI 1.057–1.243), whereas women with MetS had 23% higher risk of showing abnormal TUG result (aOR 1.226, 95%CI 1.126–1.335) in the fully adjusted model (Table 2).

**Table 2:** Association between metabolic syndrome and timed up and go test.

Metabolic syndrome	Number	Abnormal TUG	Crude	Model 1	Model 2
			OR (95% CI)	aOR (95% CI)	aOR (95% CI)
**Total**					
All	40,767	4391			
Normal	33,879	3499	Ref	Ref	Ref
Abnormal	6888	892	1.183 (1.117–1.253)	1.202 (1.134–1.273)	1.183 (1.115–1.254)
**Sex**					
*Men*					
All	20,936	1938			
Normal	17,020	1520	Ref	N/A	Ref
Abnormal	3916	418	1.159 (1.071–1.254)		1.146 (1.057–1.243)
*Women*					
All	19,831	2453			
Normal	16,859	1979	Ref	N/A	Ref
Abnormal	2972	474	1.252 (1.151–1.361)		1.226 (1.126–1.335)

Model 1: sex.

Model 2: Model 1 + depressive mood, cognitive impairment.

*TUG* timed up and go, *OR* odds ratio, *aOR* adjusted odds ratio, *CI* confidence interval, *N/A* not applicable.

### Association of each feature of metabolic syndrome with timed up and go test

For each feature of MetS, a J-shaped relationship of all features, except TG and HDL-C, with TUG was noted; TUG increased with the increase in TG up to 550 mg/dL, and then eventually decreased. For HDL-C, TUG decreased according to the increase in HDL-C up to 40 mg/dL, and the curve flattened subsequently.

**Figure 2:**
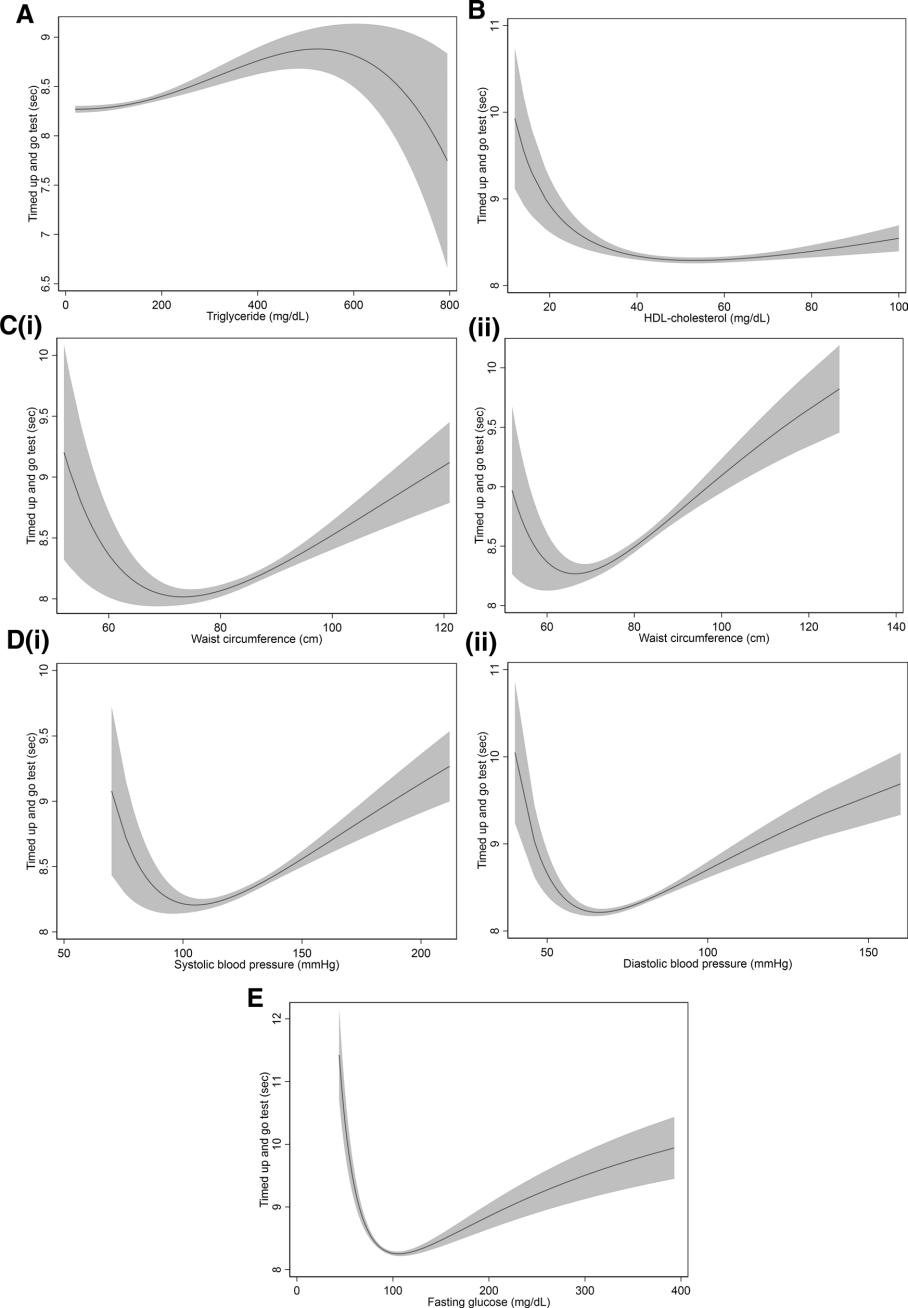
Association of Timed up and go test with features of metabolic syndrome, (**A**) TG, (**B**) HDL-C, (**C-i**) waist circumference of men, (**C-ii**) waist circumference of women, (**D-i**) systolic blood pressure, (**D-ii**) diastolic blood pressure, (**E**) fasting glucose. Gray area depicts 95% confidence interval.

Among men and women, the best TUG result was obtained when the waist circumference range was 70–80 cm and 60–70 cm, respectively. The best TUG result was achieved when systolic BP ranged between 100 and 120 mmHg and diastolic BP ranged between 65 and 75 mmHg and when fasting glucose level was approximately 100 mg/dL (Fig. 2).

### Stratified analysis

**Figure 3:**
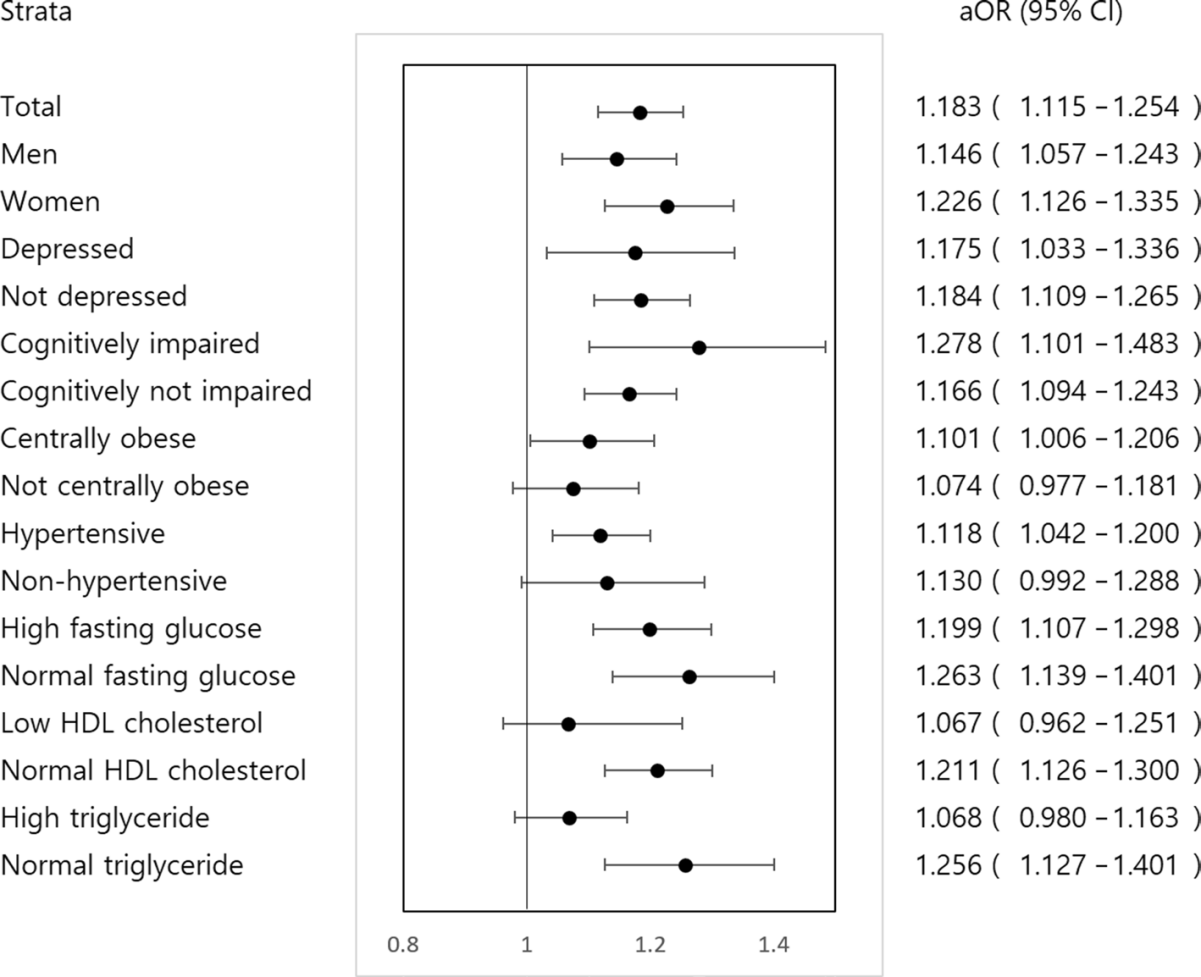
Forest plots showing stratified analyses of the association of the timed up and go test with metabolic syndrome. *aOR adjusted odds ratio.

Stratified analysis revealed that the participants with MetS were more likely to have abnormal TUG results regardless of depressive mood, cognitive impairment, or fasting glucose level. In contrast, association of TUG with MetS was significant only in participants with central obesity, high BP, normal HDL-C, and normal TG (Fig. 3).

## Discussion

This large general population-based study evaluated the association between MetS and TUG test in a nationally representative sample from an Asian country. Using a fully adjusted model, we found that MetS was associated with abnormal TUG results, and the worst TUG result was observed when participants had three or four MetS features. Furthermore, each MetS feature, except TG and HDL-C, has a J-shaped relationship with TUG. In stratified analysis, we found that the association between MetS and TUG was independent of depressive mood, cognitive impairment, or fasting glucose level, whereas the association was significant only in participants with central obesity, high BP, normal HDL-C level, and normal TG level.

A previous cross-sectional study of 83 patients with Parkinson’s disease demonstrated that participants with modifiable CV risk factors exhibited slower TUG times compared with those exhibited by participants with normal CV risk^27^. A study conducted on 18 elderly women aged > 60 years reported that TUG times of women with MetS was longer than that of women without MetS^28^, and another study of 28 elderly women also showed a similar result^29^. The findings from the current study is consistent with the results of studies that investigated the association between MetS and TUG results. However, those were small-scale studies limited to a specific population, whereas the present study used a large national representative sample comprising 66-year-old adults of an Asian country. Therefore, this study substantiated the evidence to previous studies for overcoming the limited generalizability of the relationship between MetS and TUG test. Nevertheless, because the present study has a cross-sectional design, the result cannot infer the causal relationship between MetS and TUG. A large-scale longitudinal study is warranted for evaluating this association.

The finding of the present study is in line with the studies which demonstrated that the physical performance is associated with CV disease markers. CV disease markers, including carotid artery intima-media thickness^30^, homocysteine^31^, and HDL-C^32^, were associated with walking speed. Moreover, inflammatory markers, which are risk factors for CV disease, were also associated with physical performance^33,34^. Additionally, another study reported that preclinical CV disease preceded clinical frailty^35^, suggesting that persons with impaired TUG might have had a silent preclinical vascular pathology. These findings suggest that CV risk factors and the physical performance of elderly have shared pathophysiology, such as reduction in tissue oxygenation, inflammation, and lower muscle strength and quality.

In the present study, we found that an increase up to four MetS features was associated with worse TUG result. However, when five MetS features were present, the TUG results ameliorated. There are two possible reasons for this finding. First, only 0.5% (212/40,767) of the participants showed all five MetS features, owing to which it may be possible that the TUG results of this group are subject to a high probability of random errors. This finding is depicted as a wide grey area in Fig. 1. Second, there are more men in the five MetS features group than in the other groups. There were 69.3% male participants in the five MetS features group, whereas there were 51.4% male participants in the other groups. Because men had faster TUG times than women (8.17 ± 3.06 s vs. 8.53 ± 3.08 s), it is possible that owing to the difference in sex distribution among groups, the TUG times in the five MetS features group were faster than those of the other groups.

Previous study showed that the association between physical performance and CV risk differed in men and women. Difference in sex hormone is one of possible explanations for this discrepancy. In previous studies, low testosterone level in older men was associated with falls and poorer physical performance^36^, but not significantly associated with CV risk^37^. In women, menopause is a well-known CV risk factor, and grip strength was weaker in postmenopausal women than in pre- or perimenopausal women^38^. These findings indicate that sex hormones in both sexes influence CV risk and physical performance in different ways.

Except for HDL-C and TG, all other MetS features showed a J-shaped relationship with TUG test results. This finding meant that there is an optimal range for these features with respect to their influence on mobility function. For example, fasting glucose level between 90 and 110 mg/dL could be the optimal range for an acceptable TUG result in this study population. However, TUG times increased up to a TG level of 550 mg/dL and subsequently decreased. Similar to the result concerning the number of MetS features, the aforementioned finding could be owing to a wide range of random errors and difference in sex distribution. Only 148 participants had a TG level of > 550 mg/dL, and 66% of those participants were men.

Furthermore, TUG time appears to plateau at HDL-C level of > 40 mg/dL. In a previous study conducted to investigate the association between HDL-C and lower extremity performance, HDL-C was highly correlated with knee extension torque and walking speed^32^. However, in that study, the researchers examined 4-m fast walking speed and found that there was no significant difference in the intermediate and highest tertiles in the test, whereas there were significant differences in 400-m walking speed and knee extension torque. It is likely that short distance walking tests, such as TUG or 4-m walking, are not suitable to detect the effect of HDL-C on physical performance, including pure lower extremity power or endurance.

The association between MetS and TUG results was robust regardless of some of the risk factors and MetS features. When the participants were not centrally obese or non-hypertensive, the association was not significant. This finding suggests that non-obesity and normal blood pressure have protective effects on the physical performance of participants despite them having MetS, which is consistent with the findings from previous studies^39–41^.

In a systematic review, high carbohydrate diet is associated with MetS, and replacement of carbohydrate with any type of fat, particularly polyunsaturated fat would lower TG, increase HDL-C, and lower blood pressure^42^. And high protein uptake (≥ 1.0–1.2 g/kg) was associated with better performance of lower limb^43^. In Korea, 58% of men and 60.0% of women exceeded the recommended amount of carbohydrate intake, and highest intake group showed 1.3 times higher MetS prevalence^44^, and this is high percentage of excess carbohydrate uptake compared with other countries. High carbohydrate intake in Korean older adults leads to lower percentage of protein uptake in macronutrients. In addition, while exercise is well known to have positive effects on mobility decline^45^, a previous systematic review of seven controlled trials revealed that dynamic endurance training such as walking, jogging, and cycling, which are easily performed as home-based exercise, has a favourable effects on most of MetS features^46^. Therefore, for older Korean population, replacing carbohydrate with meat, fish, egg, bean, and performing endurance exercise is needed for prevention of both MetS and mobility decline.

This study had several limitations. This study has a cross-sectional design, which prevents inference of the association between MetS and TUG to causality. Therefore, there is a need for large-scale longitudinal studies that evaluate this association. The study population included only 66-year-old men and women in Korea, which thereby prevented assessment of the association of TUG results with outcomes in different age groups. Caution should be exercised in applying the results of this study to the elderly population. The limitation of the database prevented adjustment for well-known risk factors such as household income, education, and dietary intake.

In conclusion, participants with MetS were more likely to show abnormal TUG results, and each MetS feature, except TG and HDL-C, has a J-shaped relationship with TUG. Preventive lifestyle such as lower carbohydrate and higher protein intake, and endurance exercise is needed.

## Methods

The Korean National Health Service is a public health insurance (i.e., Korean National Health Insurance Service, KNHIS) that provides universal health coverage to almost all Koreans except Medicaid beneficiaries, who account for < 3% of the total population. The KNHIS also covers the National Screening Program (NSP), which is a biennial health screening program, and the NSPTA was added to NSP in 2007. The purposes of the NSPTA were to tailor the program according to the age and sex of each subject as well as to strengthen post-examination counselling. As part of this program, only subjects aged 66 years underwent TUG test and unipedal stance tests for mobility assessment. There is no data of these tests in other ages of participants. Therefore, all participants included in the present study were 66 years old. KNHIS created the National Health Information Database (NHID), which includes data on healthcare utilisation, health screening results of the NSTPA program, sociodemographic variables, and mortality rate across 50 million participants in Korea^47^. The details of the NSPTA have been described elsewhere^48^.

### Data sources and study population

**Figure 4:**
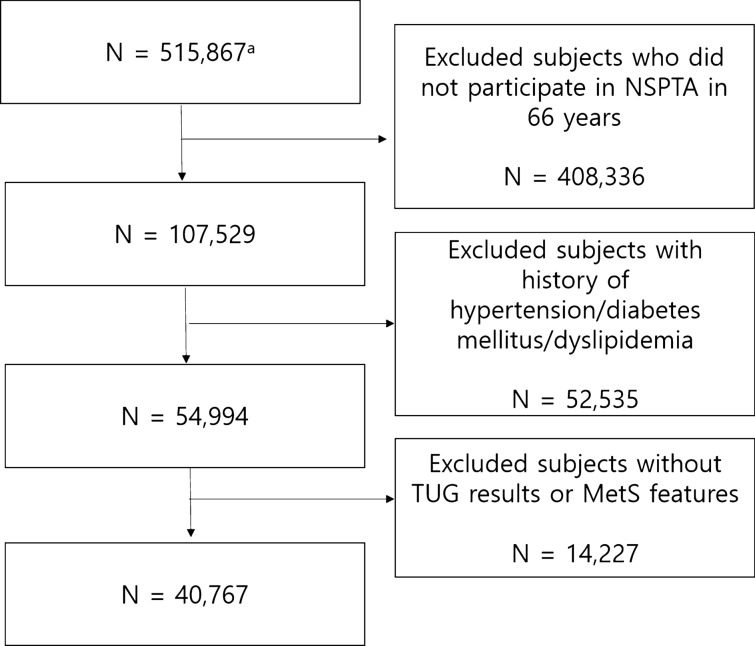
Flow of subject selection. ^a^Number of subjects in the National Health Insurance Service–National Health Screening Cohort database of the Korean National Health Insurance Service (2002–2015). *MetS metabolic syndrome, *TUG* timed up and go.

We used the National Health Insurance Service–National Health Screening Cohort database of NHID, in which 515,867 participants were included. This represents 10% of randomly selected individuals from the total Korean participants aged 40–79 years who participated in NSP at least once between 2002 and 2003. Among them, we included all 66-year-old participants who underwent NSPTA between 2007 and 2015, because the database included the data upto the end of 2015. We excluded participants with a history of hypertension/diabetes mellitus/dyslipidaemia. There are two reasons of exclusion. First, there are discrepancies between diagnostic criteria of the diseases and MetS criteria. Second, medical treatment for the chronic diseases could alter the results of laboratory tests which are not primary target of treatment, but included in MetS features. After excluding participants who already had a history of hypertension/diabetes mellitus/dyslipidaemia, those who lacked data on MetS features, those with missing data or misclassification of TUG results, or those with difficulty in performing ADL, a total of 40,767 participants were included for analysis in the current study (Fig. 4).

The database included demographic data of the participants, survey data regarding past medical history and health habits, and screening results corresponding to height, weight, abdominal circumference, physical function test (i.e., TUG test and unipedal stance test), and laboratory tests.

This study protocol was approved by the Institutional Review Board of Asan Medical Center (IRB No. 2020–0649), and this study was performed in accordance with Korean Good Clinical Practice (KGCP) guideline, which is legislated in Korea. The requirement for informed consent was waived by the Institutional Review Board of Asan Medical Center, because the KNHIS database was constructed after anonymisation according to strict confidentiality guidelines.

### Variables

#### Independent variable

##### Metabolic syndrome

The consensus definition of MetS was proposed for clinical diagnosis in 2009^49^, and we used this definition for diagnosing MetS. According to this definition, MetS could be diagnosed if patients had three or more of the following five MetS features: elevated waist circumference which was measured using the criteria of Korean Society of the Study of Obesity (≥ 90 cm) instead of the original criteria (≥ 94 cm) for men, while the cut-off for women was an ≥ 85 cm instead of the original criteria (≥ 80 cm)^50^; low HDL-C level (< 40 mg/dL); elevated TG level (≥ 150 mg/dL); elevated blood pressure (BP) (≥ 130/85 mmHg); and elevated glucose level (≥ 100 mg/dL). We excluded subjects took medications for hypertension, diabetes mellitus, or dyslipidaemia.

#### Outcome variables

##### Timed up and go test

In accordance with the procedure described in the NSPTA manual, TUG test was performed on the day of physical examination conducted as part of NSPTA at the respective community hospital where each subject was admitted. Participants were required to sit on a chair, stand, and walk a 3-m course at a comfortable speed, walk back to the chair and sit again while wearing regular footwear and/or using walking aids. The time from standing up to sitting down again was measured, with a TUG time of > 10 s categorised as abnormal. The details regarding TUG test performed as part of NSPTA has been described elsewhere^17^.

#### Potential confounders

We collected data on chronic diseases, including hypertension, diabetes mellitus, and dyslipidaemia, by administering a questionnaire. If participants answered in the affirmative for having received medications for hypertension, diabetes mellitus, or dyslipidaemia, they were considered to have the diseases and were excluded from further analysis.

Cognition was measured using the Korean Dementia Screening Questionnaire–Cognition (KDSQ-C), which is included in the NSPTA questionnaire. The KDSQ-C is a self-administered, validated questionnaire^51^, consisting of 15 items, each of which is rated on a three-point Likert scale (0, 1, or 2, with a higher score considered worse). Cognitive impairment was defined as a composite score of ≥ 6.

The NSPTA questionnaire included six items regarding ADL, which were extracted from the Korean versions of ADL (K-ADL) and Instrumental ADL (K-IADL) questionnaires^52^. The four items extracted from K-ADL were as follows: “Do you bathe by yourself without help?”; “Do you dress by yourself without help?”; “Do you eat by yourself without help if a meal is prepared?”; and “Do you go to the toilet by yourself without help?” The two items extracted from K-IADL were “Do you prepare your meal by yourself without help?” and “Do you go outside by yourself to places within walking distance?” ADL was categorised as abnormal if the answer to one or more of these questions was “No.” The procedural details to assess cognitive impairment and ADL have been described elsewhere^15^.

Depressive mood was measured using three questions extracted from the Korean Version of Geriatric Depression Scale, which has been validated elsewhere^53^. The questions were as follows: “Do you feel that your activity or desire to perform activity decreased recently?”; “Do you feel that you are useless for now?”; “Do you feel hopeless at present?” If any of these questions were answered as “Yes”, then the participants were categorised as depressed.

### Statistical analysis

With regard to baseline characteristics, continuous variables were represented as mean ± SD and categorical variables were represented as frequencies and percentages.

Multiple logistic regression models were used to evaluate the association of TUG results with MetS. Three models were built for these analyses: a crude model and two adjusted models. Model 1 was adjusted for sex, and in Model 2 depressive mood and cognitive impairment were added to Model 1. Odds ratios (OR) and 95% confidence intervals (CI) were calculated for each model.

Participants at a risk of showing abnormal TUG results were identified via stratified analyses in Model 2, with participants stratified by sex, depressive mood, cognitive impairment, and each MetS feature.

Statistical analyses were performed using STATA software (version 16.1; STATA. Corp, College Station, Texas), and a P value of < 0.05 was considered statistically significant.

### Ethics approval

This study protocol was approved by the Institutional Review Board of Asan Medical Center (IRB No. 2020–0649). Participants were informed of the purpose of research and provided written form of informed consent when they participated in NSPTA. The written consent declared that the participants understand that their information gathered through NSPTA would be used for research purpose. Administrative permission to access the NHID was acquired by National Health Insurance Sharing Service (NHIS-2018–2-201).

### Consent for publications

This paper does not include any individual person’s data in any form.

## Data Availability

The dataset generated and analysed during this study are available in National Health Insurance Sharing Service. However, the authors have no right to share or provide the data. The information of how to request for database is available in https://nhiss.nhis.or.kr/bd/ab/bdaba021eng.do. The details and cost of the database is described in https://nhiss.nhis.or.kr/bd/ab/bdaba022eng.do. To request the database, visit https://nhiss.nhis.or.kr/bd/ay/bdaya001iv.do. (only available in Korean). The questionnaire used in this study is not available in English. Korean version of the questionnaire is available for download in the following website: (http://www.law.go.kr/admRulLsInfoP.do?chrClsCd=&admRulSeq=2200000012541#AJAX).
